# Supplementary material for: Solubility of Anthraquinone Derivatives in Supercritical Carbon Dioxide: New Correlations
Source: Molecules. 2021 Jan 17;26(2):460. doi: 10.3390/molecules26020460 (PMC7831049; doi:10.3390/molecules26020460)
Supplement: Supplementary file 1 [file molecules-26-00460-s001.pdf]

Supporting Information

Solubility of Anthraquinone Derivatives in Supercritical Carbon Dioxide: New Correlations

Ratna Surya Alwi[1*], Chandrasekhar Garlapati[2], and Kazuhiro Tamura[3]

[1] Department of Chemical Engineering, Fajar University, Makassar 90231, Indonesia; ratnasya@gmail.com
[2] Department of Chemical Engineering, Puducherry Technological University (Formerly Known as Pondicherry Engineering College), Puducherry 605014, India;chandrasekar@pec.edu.
[3] Division of Natural System, Graduate School of Natural Science and Technology, Kanazawa University, Kakuma-machi, Kanazawa, 920-1192 Japan;tamura@se.kanazawa-u.ac.jp

* Correspondence: ratnasya@gmail.com; Tel.: +62-8114-480-880

Table S1 Correlation constants of Chrastil's model

Serial number & name		k	d_1	d_2
1.	C.I. disperse blue 3	4.4763	-21.857	-4041.1
2.	Blue 1	5.1932	-28.798	-5084.4
3.	1,4-dihydroxy-9,10-anthraquinone	5.3766	-24.446	-4575.3
4.	1-Hydroxy-4-(prop-2′-enyloxy)-9,10-anthraquinone	5.5655	-24.057	-5029.6
5.	1,4-bis(prop-2′-enyloxy)-9,10-anthraquinone	6.1196	-34.78	-2784.3
6.	1-amino-2-methylanthraquinone	5.3078	-26.895	-3879.4
7.	1- amino-2-ethyl-9,10-anthraquinone	5.2965	-26.017	-4187.7
8.	1-amino-2,3-dimethylanthraquinone	5.8234	-25.191	-5919.5
9.	1-hydroxy-9,10-anthraquinone	4.9491	-18.817	-5342.5
10.	1-hydroxy-2-methylanthraquinone	5.9217	-24.252	-5543.4
11.	1-hydroxy-2-(methoxy methyl)anthraquinone	5.1135	33.18	-23730
12.	1-hydroxyl-2-(ethoxy methyl)anthraquinone	5.9862	-26.315	-4885.7
13.	1-hydroxy-2-(1-propoxy methyl)anthraquinone	6.4018	-26.625	-5553.8
14.	1-hydroxy-2-(1-butoxymethyl) anthraquinone	6.5749	-27.681	-5504.4
15.	1-hydroxy-2-(n-amyloxy methyl) anthraquinone	7.3635	-34.239	-5052.7
16.	Quinizarin	6.218	-17.712	-7527
17.	Violet 1(1,4-diaminoanthraquinone)	5.965	-23.704	-7181.2
18.	Blue 59 (1,4-bis (ethyl amino)anthraquinone)	6.6818	-27.63	-7387.4
19.	Red 15 (1-amino-4-hydroxyanthraquinone)	5.6911	-22.347	-6228.3
20.	1 hydroxy-4-nitroanthraquinone	5.5724	-23.918	-6340.1
21.	1,8-dihidroxy-4,5-dinitroanthraquinone	4.5775	-23.672	-4801.5
22.	1,4 diamino-2,3-dichloroanthraquinone	6.0815	-26.323	-6851.5
23.	1-aminoanthraquinone	6.0211	-24.837	-6533.9
24.	1-nitroanthraquinone	4.7142	-20.351	-5253.7
25.	C.I. Disperse orange 11	5.4894	-23.799	-5692.1

Table S2 Correlation constants of Adachi- Lu model

Sl.No*	e_1	$e_2 \cdot 10^4$	$e_3 \cdot 10^7$	e_4	e_5
1	3.2130	8.44	-10.00	-25.0100	-2503.2
2	0.2681	31.10	-17.50	-5.0509	-4438.4
3	0.3839	12.00	-1.80	6.2903	-5022.8
4	0.4337	17.40	-5.10	6.6452	-5589.7
5	0.1506	31.00	-14.70	-3.7912	-2574.8
6	0.4846	12.90	-2.56	2.0969	-4160.0
7	1.9395	20.00	-10.30	-8.0682	-4149.6
8	0.6419	31.20	-15.50	-0.0466	-5505.9
9	0.2796	9.50	-0.78	9.3111	-5355.0
10	0.2714	21.60	-7.92	7.0457	-5545.4
11	0.1250	12.80	-3.88	50.6230	-22316.0
12	0.3027	19.30	-6.54	6.0388	-4896.3
13	0.5936	21.20	-7.67	6.0163	-5535.0
14	0.0393	11.70	-0.08	12.5920	-5813.8
15	0.4283	31.90	-15.10	-5.6222	-4898.6
16	1.2008	40.00	-29.20	0.7280	-7937.3
17	1.0884	17.70	-3.67	3.6929	-7968.1
18	2.8592	14.30	-3.75	-3.8538	-8139.0
19	0.3158	23.10	-7.58	7.9874	-7338.2
20	-0.2560	27.20	-11.40	8.2618	-6674.7
21	0.4407	8.27	1.92	3.0107	-5502.6
22	0.1546	28.50	-12.90	5.9852	-6998.7
23	0.4361	24.40	-9.41	6.4838	-6962.1
24	0.2707	21.30	-7.87	5.6946	-6074.8
25	-3.4638	43.30	-19.50	23.8160	-5998.7

Sl.No*: Serial number & name same as Table S1.

Table S3 Correlation constants of Mitra – Wilson model

Sl.No*	h_1	$h_2 \cdot 10^2$	$h_3 \cdot 10^6$	h_4	$H_5 \cdot 10$
1	8.891	-1.36	5.39	-11.501	-47.294
2	3.761	-6.53	113.00	-16.065	-3.815
3	5.303	-4.22	70.50	-11.610	-13.148
4	5.723	-7.20	127.00	-18.240	-4.810
5	7.459	-11.90	168.00	-24.747	2.502
6	6.208	-7.37	116.00	-17.935	-7.409
7	8.092	-10.50	168.00	-26.528	-5.186
8	4.704	-3.11	75.10	-11.697	-15.519
9	4.389	-5.72	116.00	-15.887	-3.196
10	6.982	-7.79	133.00	-20.730	-7.950
11	23.787	-21.30	589.00	-120.240	-32.893
12	6.021	-7.35	124.00	-18.354	-4.578
13	4.808	-5.94	115.00	-15.907	-3.213
14	5.696	-7.79	146.00	-20.068	-1.074
15	6.435	-10.00	181.00	-25.430	-5.120
16	10.154	-7.60	151.00	-28.579	-19.051
17	5.159	-6.70	149.00	-19.396	-9.266
18	17.996	-11.70	176.00	-43.868	-45.536
19	5.135	-8.79	196.00	-24.666	0.166
20	4.634	-6.22	136.00	-17.711	-6.775
21	2.974	-3.91	89.00	-10.332	-8.543
22	17.718	-7.33	81.30	-32.425	-60.379
23	11.412	-9.71	172.00	-32.929	-23.037
24	4.654	-3.07	77.90	-9.339	-17.738
25	12.879	-14.90	258.00	-45.414	-11.446

Sl.No*: Serial number & name same as Table S1.

Table S4 Correlation constants of Keshmiri et al. model

Sl.No*	f_1	f_2	$f_3 \cdot 10^5$	f_4	f_5
1	42.838	-33867.00	-1.60	-5.1045	4146.20
2	-130.410	32136.00	-0.12	19.4580	-5580.20
3	-27.977	-2968.30	0.08	4.8363	-221.73
4	-34.836	-929.29	0.20	6.0491	-579.50
5	-65.400	7677.90	-0.13	9.7406	-1573.50
6	-50.287	4834.20	0.27	7.5598	-1231.30
7	-37.878	-1324.00	-0.27	6.3393	-506.46
8	-56.428	5983.30	0.17	9.2355	-1699.60
9	-54.382	10024.00	0.45	8.6146	-2098.70
10	-55.625	5926.80	0.16	9.3832	-1645.30
11	64.673	-12390.00	7.69	-12.3240	1887.40
12	-122.180	30075.00	0.39	18.8260	-5079.10
13	-55.223	5782.90	0.24	9.4094	-1615.60
14	-93.677	18840.00	0.40	15.0030	-3510.50
15	-11.505	-13949.00	-0.18	3.1955	1260.00
16	-57.946	7823.00	-0.02	11.5840	-2422.90
17	3.984	-15642.00	1.31	-0.3851	1556.30
18	-62.481	6015.30	0.95	10.6630	-1952.20
19	-32.072	-2871.30	0.65	5.7151	-457.79
20	-40.739	3121.30	1.55	6.1651	-1148.00
21	-63.318	11554.00	1.64	-4.8200	-2302.30
22	-63.104	4304.40	-0.93	-2.1996	-1893.40
23	-35.609	-2202.10	0.59	-0.4448	-597.06
24	-41.603	2118.70	0.05	-7.4179	-1155.10
25	48.553	-31793.00	-0.73	-6.1787	3872.90

Sl.No*: Serial number & name same as Table S1.

Table S5 Correlation constants of Khansary et al. model

Sl.No*	l_1	l_2	$l_3 \cdot 10^2$	l_4	$l_5 \cdot 10^2$
1	-7130.00	-0.1740	-4.010	1.0900	3.270
2	-11514.00	-0.2114	1.108	3.5414	2.736
3	-11788.00	-0.2081	-0.249	4.2345	3.032
4	-6357.60	-0.1490	-0.490	1.4736	2.304
5	-7190.00	-0.1690	-0.312	2.0000	2.540
6	-6465.90	-0.2270	-0.639	1.6318	3.451
7	-5894.80	-0.1723	-0.424	1.2020	2.627
8	-7766.20	-0.2082	-0.432	2.1852	3.103
9	-9819.00	-0.2228	-0.039	3.1253	3.220
10	-9256.10	-0.1333	-0.482	1.7036	2.151
11	-9351.10	-0.1482	-1.574	1.4338	2.604
12	-3765.50	-0.0721	-0.946	0.1069	1.313
13	-6982.70	-0.1543	-0.509	1.9131	2.379
14	8680.00	-0.1050	-37.400	-9.3200	6.790
15	-5767.60	-0.1561	-0.534	1.4001	2.415
16	-6763.60	-0.1440	-0.442	1.9090	2.226
17	-6104.80	-0.1506	-0.518	1.6116	2.346
18	-8570.00	-0.2550	-0.494	3.0000	3.800
19	-14654.00	-0.1528	-1.192	5.9729	2.309
20	2224.70	-0.0749	-11.506	-5.9342	3.566
21	-8988.00	-0.1722	-2.521	1.8814	3.019
22	-8679.60	-0.1520	0.201	2.2823	2.232
23	-7922.40	-0.1450	-0.487	1.5834	2.291
24	-5740.70	-0.1276	-0.627	0.2554	2.069
25	-13025.00	-0.1996	0.954	4.4551	2.674

Sl.No*: Serial number & name same as Table S1.

Table S6 Correlation constants of Bian et al. model

Sl.No*	g_1	g_2	g_3	g_4	$g_5 \cdot 10^3$
1	-30.382	-5254.60	4.19	5.7610	-2.490
2	-52.597	5763.90	-25.09	4.6141	9.190
3	-39.907	2038.50	-13.37	5.2654	4.100
4	-59.575	4875.90	-24.77	7.9383	7.890
5	-71.348	9996.10	-34.77	7.2843	13.100
6	-57.634	5753.20	-25.23	6.8066	8.610
7	-61.820	3648.40	-19.37	9.0028	4.210
8	-43.057	2029.80	-17.26	5.6837	5.580
9	-33.485	1387.50	-13.85	4.5272	4.470
10	-59.537	5474.80	-26.90	7.7626	8.900
11	-157.040	29478.00	-63.82	13.6170	22.500
12	-53.771	5122.30	-24.07	6.7470	8.240
13	-42.078	3474.40	-19.92	5.3085	7.180
14	-54.842	6228.60	-30.11	6.5167	11.300
15	-58.359	-3064.10	-2.23	9.6368	0.307
16	-87.007	8431.30	-45.67	13.0940	13.200
17	-66.595	5060.70	-28.85	8.3958	9.420
18	-69.012	5478.20	-29.81	9.0056	9.660
19	-92.904	5880.40	-32.40	14.3170	6.540
20	-55.474	4476.70	-25.16	6.5729	8.880
21	-30.194	1431.50	-10.10	2.2873	4.860
22	-96.950	6074.00	-34.09	14.7870	6.910
23	-77.993	2352.90	-17.12	13.0430	0.541
24	-51.928	4226.80	-24.06	6.1810	8.600
25	-44.853	-5204.70	12.97	9.7842	-10.400

Sl.No*: Serial number & name same as Table S1.

Table S7 Correlation constants of Garlapati – Madras model

Sl.No*	i_1	i_2	$i_3 \cdot 10^4$	i_4	$i_5 \cdot 10^2$
1	67.79	-3.158	-3.207	12600.00	1.876
2	-60.65	18.665	-20.910	-14024.00	-1.200
3	-83.99	-9.508	8.364	19373.00	3.402
4	-70.31	-13.347	10.610	21217.00	3.641
5	-90.04	-8.812	3.330	20093.00	3.506
6	-102.63	-7.850	2.486	22005.00	3.536
7	-75.28	-10.723	7.559	19697.00	3.321
8	-110.19	-6.925	-2.191	22096.00	3.655
9	-131.19	-2.571	-7.110	20039.00	3.647
10	-74.21	-13.309	7.677	22176.00	3.770
11	-87.87	-8.928	4.163	19703.00	3.507
12	-95.69	-13.196	-4.134	17426.00	5.525
13	-71.63	-11.431	9.521	19414.00	3.452
14	-46.39	-14.438	15.950	17696.00	3.321
15	-87.40	-12.634	8.445	23227.00	4.069
16	-72.37	0.297	0.014	7740.20	1.807
17	-96.17	-5.580	-2.928	19528.00	3.190
18	-39.63	-3.868	9.545	4307.70	1.521
19	-81.88	-11.517	11.320	20196.00	3.608
20	-79.56	-12.214	10.640	21299.00	3.599
21	-67.99	-11.445	12.050	18372.00	3.166
22	-44.39	-9.846	12.630	12799.00	2.283
23	-87.82	-9.917	5.355	20690.00	3.411
24	-76.12	-11.560	10.600	19921.00	3.455
25	-78.68	-12.096	8.989	21932.00	3.479

Sl.No*: Serial number & name same as Table S1.

Table S8 Correlation constants of Reddy et al. model

Sl.No*	J_1	J_2	J_3	J_4	J_5
1	2.100	-0.190	-8.120	1.350	-9.130
2	-3.664	3.217	-3.488	-3.380	-8.315
3	-12.685	3.253	15.677	-3.060	-13.223
4	6.104	4.022	-28.289	-3.765	11.965
5	1.242	8.624	-43.855	-8.555	33.586
6	-17.124	4.998	17.245	-4.816	-10.901
7	-8.391	6.991	-11.203	-6.943	9.151
8	-1.259	1.856	1.784	-1.501	-13.471
9	-4.438	4.684	-5.537	-4.618	0.663
10	-0.056	5.749	-20.750	-5.601	11.689
11	0.577	0.415	3.030	-3.610	-8.030
12	-5.700	4.085	-1.399	-3.863	-1.952
13	-1.832	3.765	-7.029	-3.484	-0.104
14	3.102	5.034	-23.922	-4.803	12.434
15	-86.400	7.960	150.000	-7.840	-71.900
16	-4.388	0.078	-8.505	0.949	6.544
17	-0.471	3.881	-12.957	-3.199	-2.786
18	-4.491	4.512	-6.482	-3.693	-4.032
19	0.095	-0.127	-0.856	1.711	-16.212
20	-1.134	3.378	-9.356	-2.789	-4.119
21	-2.190	0.658	1.975	0.105	-16.694
22	2.351	2.173	-13.060	-1.055	-6.412
23	1.085	3.327	-14.510	-2.408	-0.812
24	-2.206	1.116	0.105	-0.061	-12.895
25	4.372	3.642	-12.201	1613.400	-3.456

Sl.No*: Serial number & name same as Table S1.
